# Comparative efficacy of two different topical povidone-iodine 5% regimens in reducing conjunctival bacterial flora: A randomized parallel double-masked clinical trial

**DOI:** 10.1371/journal.pone.0189206

**Published:** 2017-12-19

**Authors:** Letícia Fernandes Barroso, Sarah Pereira Cazella, Antonio Brunno Nepomuceno, Luiza Toscano, Liliane Ângela de Souza Castilho, Eloísa Marcela Rueda Furlan, André Messias, Ingrid U. Scott, Rodrigo Jorge

**Affiliations:** 1 Department of Ophthalmology, Otorhinolaryngology and Head and Neck Surgery, School of Medicine of Ribeirão Preto, University of São Paulo–USP, Ribeirão Preto, São Paulo, Brazil; 2 Department of Ophthalmology, Penn State College of Medicine, Hershey, PA, United States of America; 3 Department of Public Health Sciences, Penn State College of Medicine, Hershey, PA, United States of America; Poznan City Hospital, POLAND

## Abstract

**Introduction:**

The increasing prevalence of multi-resistant bacteria is a major public health concern. Infections acquired during ophthalmic surgery are devastating. The purpose of the current study is to compare the proportion of eyes with negative bacterial cultures on all tested media after 1 versus 3 sequential drops of povidone-iodine (PI) 5% into the inferior conjunctival fornix.

**Methods:**

Patients were randomly assigned to receive 1 (PI group) drop (at time 28 minutes) or 3 (PI plus group) sequential drops (at time 0, 20 minutes and 28 minutes) of PI 5% into the inferior conjunctival sac of one randomly selected eye. A swab culture was obtained from the inferior conjunctival fornix 5 minutes before and 30 minutes after time 0. Central corneal thickness (CCT) was measured shortly before time 0 and shortly after time 30. Conjunctival swabs were incubated aerobically in enriched Thioglycolate liquid medium (meat broth) and in three solid culture media (chocolate agar, trypticase soy agar with 5% sheep blood, and Sabouraud agar).

**Results:**

There was no significant difference in the proportion of negative cultures after intervention between groups (p = 0.1638). Also in the PI group (n = 59), the proportion of eyes with negative cultures after PI (79.7%) did not differ significantly from baseline (76.3%; p = 0.7539). However in the PI plus group (n = 61), the proportion of eyes with all negative cultures after PI (85.3%) was significantly higher than before PI (70.5%) (p = 0.0177). There was no significant difference in mean CCT before and after the intervention in both groups.

**Conclusion:**

Instillation of 3 sequential drops of PI was associated with a significant increase in the proportion of eyes with all negative cultures, while instillation of a single drop of PI was not associated with a significant increase in the proportion of negative cultures. Further study is warranted to determine whether the difference between the PI administration regimens is also associated with differences in the rates of postoperative ocular infections.

## Introduction

Studies have demonstrated that conjunctival and eyelid flora are the most common sources of postoperative endophthalmitis.[1,2] In 82% of patients with endophthalmitis from whom microorganisms were isolated from the vitreous fluid, the bacteria were genetically indistinguishable from bacteria isolated from the eyelid, conjunctiva or nose of the patient.[1,2]

The objective of antisepsis is to significantly reduce the number of microorganisms in the surgical field.[2] It is believed that reducing the number and growth of bacteria on the ocular surface and adnexa reduces the risk of postoperative endophthalmitis.[3]

Topical application of gatifloxacin for 3 days significantly enhances the proportion of eyes with negative bacterial cultures on all tested media compared to untreated eyes.[4] However, after instillation of PI, there was no significant difference in the proportion of eyes with negative bacterial cultures on all tested media between the untreated eyes (control) and eyes pretreated with topical gatifloxacin. These results suggest that the use of topical gatifloxacin in combination with PI for 3 days does not induce a greater reduction of bacterial colony counts than preoperative instillation of PI alone. Further, PI has not been associated with the development of antimicrobial resistance or the presence of more virulent species of bacteria, both of which have been reported in association with use of topical antibiotics.[3,5]

The optimal number of PI drops, and optimal duration of PI contact on the ocular surface, to reduce ocular surface bacterial flora prior to ophthalmic surgery have not been established.[6] Although inexpensive and probably effective, a larger quantity of PI and/or longer duration of PI contact may be toxic to the ocular surface. The potential for ocular toxicity and the concerning emergence of resistant multi-resistant bacteria provide a strong rationale for detailed study of existing antiseptic methods.[7]

The purpose of the current study is to compare the proportion of eyes with negative bacterial conjunctival cultures on all tested media after instillation of 3 sequential drops of PI 5% over 28 minutes to the instillation of 1 drop of PI 5% into the inferior conjunctival fornix.

## Materials and methods

The study protocol adheres to the tenets of the Declaration of Helsinki and was approved by the local Institutional Review Board and Ethics Committee of the Hospital das Clínicas, University of São Paulo, Ribeirão Preto, Brazil (2516/2010) on August 3^rd^ 2010. The study is registered at www.clinicaltrials.gov (NCT01739920). All participants gave written informed consent before entering into the study.

Consecutive eligible patients evaluated in the Retina Section of the Department of Ophthalmology, School of Medicine of Ribeirão Preto (HC-FMRP-USP), São Paulo, Brazil between May 2011 and October 2014, were invited to participate in this double-masked randomized clinical trial. The Ethics Committee of the Hospital das Clínicas, University of São Paulo, Ribeirão Preto, Brazil adheres to Plataforma Brasil and makes available online study protocols at the time of protocol acceptance. The authors confirm that all ongoing and related trials for this drug/intervention are registered. All protocol changes were approved by the Ethics Committee of the Hospital das Clínicas, University of São Paulo, Ribeirão Preto, Brazil.

### Patient eligibility

#### Inclusion criteria

1) Age 18 years or older; 2) no systemic or ocular infection; 3) absence of autoimmune disease or immunosuppressive therapy; 4) no use of systemic or topic antibiotics in the preceding 30 days; 5) no previous ocular surgery or trauma in the study eye in the last 30 days; 6) no history of allergy to PI; 7) signed informed consent.

#### Exclusion criteria

1) Presence of blepharitis, ectropion, entropion, trichiasis or distichiasis; 2) diabetes mellitus; 3) any systemic condition that, at the discretion of the investigator, would predispose to infection, such as influenza; 4) inability to understand and/or unwilling to provide informed consent.

### Treatment assignment

One eye of each patient was randomly assigned to one of the two following study groups:

#### PI plus group

Patients in the PI plus group underwent a baseline slit-lamp examination and had their central corneal thickness (CCT) measured by ultrasonic pachymeter (OcuScan Alcon RXP, Fortworth, USA). A sterile conjunctival swab (Cathc-All^TM^, Sample Collection Swab, Madison, USA) was used to take a culture of the conjunctiva 5 minutes before the first drop of PI 5% was instilled into the inferior conjunctival sac of the study eye. The time of the first PI 5% eye-drop instillation was considered time 0. At 20 minutes and at 28 minutes, a second and third PI 5% eye-drop, respectively, were administered into the inferior conjunctival sac of the study eye. At 30 minutes, a second conjunctival sac swab was obtained. At 35 minutes, CCT and slit-lamp examination were again performed to investigate for evidence of corneal epithelial toxicity.[8] Swabs were rotated completely 5 times with slight pressure against the inferior conjunctival fornix without contacting the other eye or any skin structure. Sampling was performed, using sterile gloves and a face mask, by the same researcher for all patients.

#### PI Group

Patients underwent the same procedures and swabs as patients in the PI plus group, except that the eye-drops administered at time 0 and at 20 minutes consisted of sterile saline solution 0.9% instead of PI 5% solution.

Before the application of any eye-drop (PI or saline) or any conjunctival swab, 1 drop of proximetacaine (Visonest®, Alcon, FortWorth, USA) was administered to the study eye in both groups.

### Microbiology assay

The samples obtained from conjunctival swabs were sent to the Microbiology Laboratory, University Hospital of Ribeirão Preto Medical School, where they were incubated aerobically in enriched Thioglycolate liquid medium (meat broth) and in three solid culture media (Agar Chocolate, Trypticase Soy Agar with 5% sheep blood, and Agar Sabouraud). The Agar Sabouraud medium was incubated at 27°C. Agar Chocolate, Agar Blood and Thioglycolate media were incubated at 35 to 37°C. The cultures were examined daily starting 24 hours after seeding and up to 72 hours. The liquid Thioglycolate medium was sowed again as soon as it showed turbidity or after five days of incubation without turbidity. Sodium thiosulfate (0.5%) was added to the enriched Thioglycolate medium in order to neutralize the antiseptic iodine-povidone. [9,10] Any microorganism that grew in the respective culture media was then isolated and identified with the automated Vitek 2 Technology system (bio-Mérieux®, Marcy-l'Étoile, France). Tests to detect sensitivity to antibiotics were carried out using E-Test and the Vitek 2 Technology automated system with Advanced Expert System, which analyzes minimum inhibitory concentration patterns and detects the phenotype of the different microorganisms tested, following the criteria established by the Clinical and Laboratory Standards Institute.

### Outcome measures

The primary outcome measure is the proportion of eyes with all negative bacterial cultures of the conjunctiva on all tested media after PI administration. Secondary outcomes include: 1) the proportion of eyes with negative bacterial cultures of the conjunctiva after PI administration adjusted by values before PI; 2) difference between the two PI groups with respect to change in CCT after PI administration compared to before PI.

### Statistical analysis

Inter-group analysis was performed to compare the PI and PI plus groups with respect to the proportion of eyes with negative bacterial cultures on all tested media. Intra-group analysis was performed to compare the proportion of eyes with negative cultures on all tested media before and after PI instillation in each group.

For the inter-group analysis, the relative risk (RR) of achieving the primary outcome (all negative bacterial cultures after PI) was calculated (unadjusted inter-group analysis). We also used a generalized linear model (GLM) with logit binary function (adjusted inter-group analysis).[11] In GLM, the results obtained after treatment were considered as a dependent variable, the type of treatment as the independent variable and culture results before intervention as a covariate. This model resulted in an odds ratio (OR) for the comparison of the proportion of eyes with negative bacterial cultures on all tested media between the PI and PI plus groups adjusted by the proportion of eyes with negative bacterial cultures on all tested media before any intervention.

Comparisons of the intra-group results were performed using the McNemar exact test and OR for paired data.[12] The mean post-treatment pachymetry results were compared between the pre-treatment results using a covariance model analysis (ANCOVA). Missing values were not included in the statistic analysis.

Statistical significance was defined by p<0.05 and by a 95% confidence interval (CI). The analysis was performed using SAS v 9.4 (SAS Institute Inc., 2012). Losses related to the results of examinations were ignored and those patients were excluded (per-protocol analysis).

### Sample size

Sample size calculation was performed based on previously published data and on a pilot study performed at our institution that demonstrated an increase of 20% in the number of negative cultures with the use of 3 PI drops (90% all negative cultures was found in the pilot study for PI plus) compared to the use of only 1 PI drop (70% all negative cultures was found in the pilot study for PI). The formula proposed by Sakpal 2010 for the calculation of sample size in clinical trials [13] was used. We considered the use of a two-tailed test with 80% power and a 5% level of significance. A sample size of 118 eyes, 59 in each arm, was considered sufficient to detect a difference of 20% in the number of negative cultures between groups. The results of the pilot study were analysed after protocol approval and before patient enrollment.

### Randomization

A block randomization was performed using a fixed number of 4 participants with random distribution within the blocks. A randomization list was generated and remained in possession of HC-FMRP-USP staffs, who were not related to the inclusion of patients and evaluation of outcome in order to protect allocation concealment until the intervention was assigned. After patient allocation, the study eye was selected using a simple randomization method (flipping a coin).

### Masking

A sham intervention was performed by the substitution of the first two PI drops by sterile saline 0.9% in the PI group. The application of the intervention was carried out by a physician not related to patient enrollment, randomization and outcome assessment. Recruiters, patients and outcome assessors were not informed of the PI administration regimen performed in each patient.

## Results

**Fig 1:**
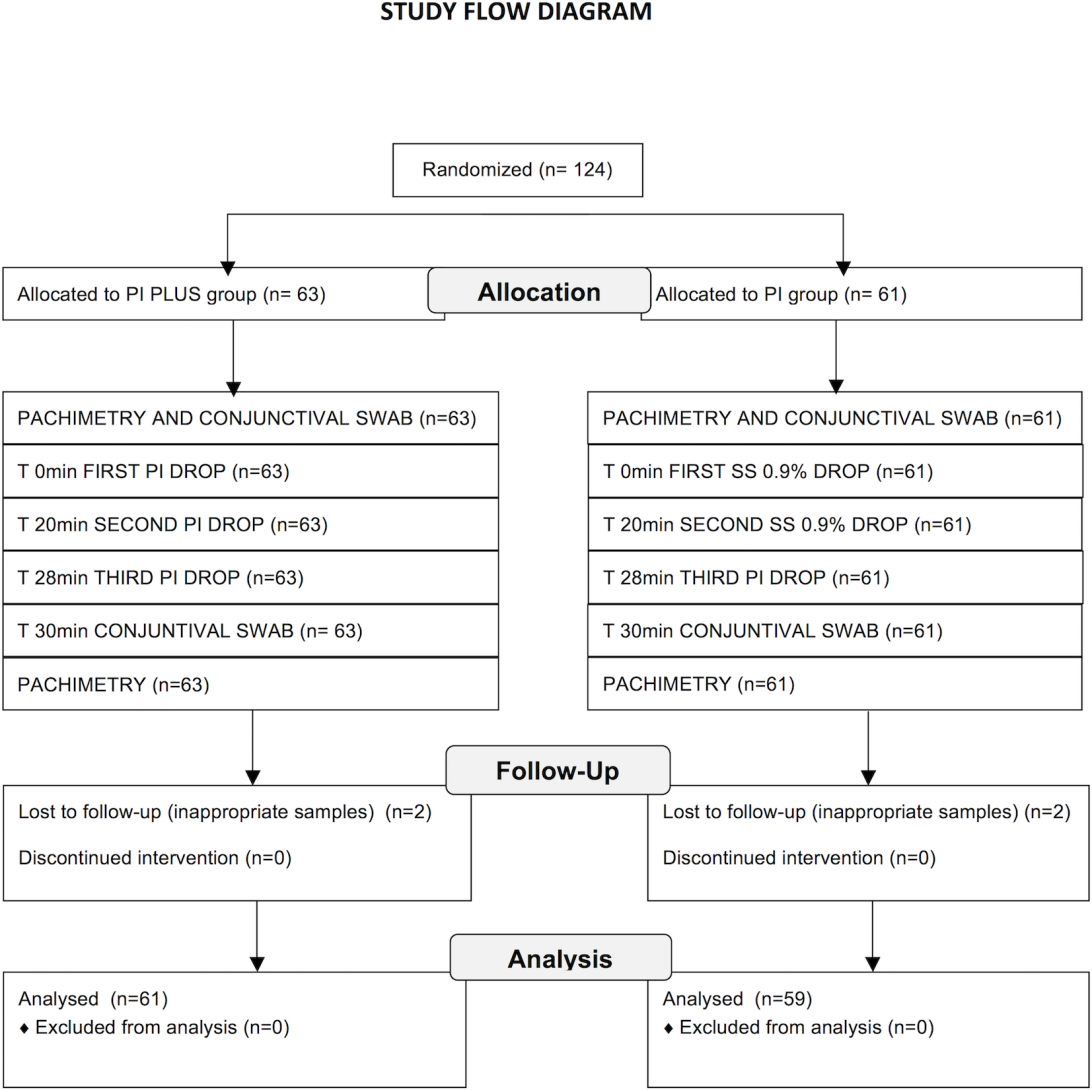
Flow diagram of the study. T = time; PI = povidone-iodine 5%; SS = saline solution.

The study population consists of 124 patients, including 61 in the PI group and 63 in the PI plus group (Fig 1). Table 1 summarizes the patient demographic and baseline characteristics. Culture results are unknown for two patients in each group due to inadequate sample (Fig 1). Those patients were excluded from the analysis.

**Table 1:** Patient demographic data and baseline characteristics.

	GROUP	
Variables	PI	PI plus
**Gender: n (%)**		
Female	37 (60.66)	31 (49.20)
Male	24 (39.35)	32 (50.80)
**Mean age in years**	63.90	62.34
**(Standard deviation)**	(14.02)	(14.46)
**Mean CCT**^**a**^ **before intervention (um)**	521.51	522.97
**(Standard deviation)**	(33.75)	(36.04)
**Positive culture before intervention (n)**	6	10

^a^CCT = central corneal thickness

The number of eyes with negative cultures on all tested media in each group before and after treatment is displayed in Table 2. The most commonly isolated pathogen was *Staphylococcus epidermidis*, cultured from 9 eyes before intervention and 9 eyes after intervention in the PI group; and cultured from 8 eyes before intervention and 5 eyes after intervention in the PI plus group. Other bacteria species were significantly heterogeneous.

**Table 2:** Intra- and inter-group comparison of culture results.

Intra-group analysis†										Inter-group analysis^‡^
				OR (95% CI)^#^					OR (95% CI)^#^	OR (95% CI)
	PI group			p-value*		PI plus group			p-value*	p-value
	+	-	Total			+	-	Total		
Intervention	after	after	n (%)		Intervention	after	after	N (%)		
**+**	8	6	14 (23.7)	1.5 (0.4–7.2)	**+**	8	10	18 (29.5)		
**before**					**before**				10.0 (1.4–434.0)	2.2 (0.7–6.8)
**-**	4	41	45 (76.3)	0.7539	**-**	1	42	43 (70.5)		
**before**					**before**				0.0117	0.1638
**Total n (%)**	12 (20.3)	47 (79.7)	59 (100.0)		**Total n (%)**	9 (14.7)	52 (85.3)	61 (100.0)		

+ = number of positive results

- = number of negative results.

(*)The exact version of Mc Nemar test was used for intra-groups comparisson.

(#) Odds ratio (OR), calculated for paired data.

(†) For the intra-groups comparisson 0.5 was added to each cell for the OR calculation.

(‡) The inter-groups comparisson was calculated using a Generalized linear model.

Resistance to at least one of the tested antibiotics was found in a significant proportion bacteria isolated before or after intervention (Table 3). The highest proportion of bacterial resistance was to the beta-lactam antibiotics. Quinolone-resistant organisms were isolated from 2 eyes in the PI group and 4 eyes in the PI plus group (Table 3). Methicillin-resistant *Staphylococcus epidermidis* was isolated from 5 eyes in the PI group and 6 eyes in the PI plus group. Methicillin-resistant *Staphylococcus aureus* was isolated from one patient in each group. No vancomycin resistant organisms were detected in any of the samples.

**Table 3:** Bacterial resistance in different antimicrobial classes.

	Group		
	PI	PI plus	
	n (%)	n (%)	p-value*
Resistance to at least one antibiotic			
Susceptible	5 (27.78)	4 (21.05)	0.7140
Resistant	13 (72.22)	15 (78.95)	
Beta-lactam			
Susceptible	8 (44.44)	6 (33.33)	0.7332
Resistant	10 (55.56)	12 (66.67)	
Methicillin (*Staphylococcus spp*.)			
Susceptible	9 (56.25)	7 (50.00)	1.0000
Resistant	7 (43.75)	7 (50.00)	
Ceftazidime			
Susceptible	18 (100.00)	18 (94.74)	1.0000
Resistant	0 (0.00)	1 (5.26)	
Quinolones			
Susceptible	16 (88.89)	15 (78.95)	0.6599
Resistant	2 (11.11)	4 (21.05)	
Moxifloxacin			
Susceptible	16 (88.89)	16 (84.21)	1.0000
Resistant	2 (11.11)	3 (15.79)	
Aminoglycosides			
Susceptible	11 (68.75)	15 (78.95)	0.7003
Resistant	5 (31.25)	4 (21.05)	

*Fisher exact test

### Unadjusted inter-group analysis

Forty-seven of 59 patients in the PI group (79.7%) and 52 of 61 patients in the PI plus group (85.3%) showed a negative culture after intervention. The univariate RR of this comparison was 0.9345 (p = 0.4234; 95% CI = 0.7916 to 1.103). The univariate OR was 0.6779 (p = 0.4225; 95% CI = 0.2622 to 1.753).

### Generalized linear model (GLM)–adjusted inter-group analysis

There was no significant difference comparing values of culture results after intervention, considering culture results before intervention for adjustment p = 0.1638, OR = 2.2 (95%CI = 0.7–6.8).

### Intra-group analysis

In the PI group, the proportion of eyes with negative bacterial cultures on all tested media after PI (79.7%) did not differ significantly from baseline (76.3%) (p = 0.7539; OR = 1.5, 95% CI: 0.4 to 7.2) (Table 2). In the PI plus group, the proportion of eyes with negative bacterial cultures on all tested media was significantly higher after PI (85.3%) than before PI (70.5%) (p = 0.0177; OR = 10.0, 95% CI: 1.4 to 434.0). In the PI plus group, patients were 10 times more likely to have all negative bacterial cultures after PI than before PI.

### Central corneal thickness (CCT) evaluation

There was no significant difference between the PI and PI plus groups with respect to the mean change in CCT after PI compared to baseline (Table 4). There was also no difference between the groups in mean CCT after PI, when adjusted for pachymetry values before treatment (p = 0.5228) (Table 4).

**Table 4:** Central corneal thickness (CCT) before and after intervention.

	Mean Central Corneal Thickness (um)		Inter-group comparison	
			Diference (PI-PI plus)	
	PI	PI plus	(CI 95%)	p-value*
**CCT**				
Before intervention (95% CI)	521.51 (512.71–530.30)	522.97 (513.74–532.20)		
After intervention (95% CI)	522.85 (513.36–532.33)	522.57 (511.68–533.46)		
Difference (before-after); (p-value)^†^	1.34 (-2.30–4.98); (0.4641)	-0.39 (-4.66–3.88); (0.8544)		
After intervention (95% CI) (adjusted)^#^	523.62 (519.66–527.59)	521.82 (517.92–525.72)	1.80 (-3.76; 7.36)	0.5228

(†) Intra-group comparison (before treatment x after treatment) were calculated using a paired t test.

(*) Statistical calculation for comparison between PI and PI plus groups were calculated using ANCOVA.

Values before treatment were used for adjustment (covariate).

(#) Mean values were adjusted using basal values from the ANCOVA model.

## Discussion

The increasing prevalence of multi-resistant bacteria is a significant public health concern.[7,14,15] Such bacteria are commonly found in hospital environments, in patients who have undergone multiple surgical procedures and in patients hospitalized for a long period. [16,17] Preventable factors contributing to the emergence of virulent multi-resistant micro-organisms include irresponsible use of antibiotics and inadequate antiseptic techniques.[18] Recently, the growing number of indications for intravitreal injections has resulted in a growing number of endophthalmitis cases after ophthalmologic procedures.[3]

The potentially devastating consequences of endophthalmitis and the relatively low penetration of most systemic antibiotics into the eye justify the need for studying how to optimize antiseptic procedures before invasive ocular procedures. Prior studies show that PI acts as an effective antiseptic agent prior to intraocular surgery.[19]

The proportion of eyes with negative bacterial cultures on all tested media before antiseptic treatment varied from 70.5 to 76.3% in the PI plus and PI group, respectively. In a previous study, 50.7 to 62.8% of patients had negative cultures on all tested media in the contralateral eyes of patients undergoing intravitreal injections before any antiseptic treatment.[4] Other studies reported lower rates: 28/272 (10.3%) patients selected for a prospective study of different anti-septic regimens had all negative cultures before any treatment.[20] The difference across various studies in the proportion of patients with all negative ocular surface cultures may be related to different microbiology techniques employed for conjunctival microorganisms culture: in the latter study, a specific medium developed at Research Foundation for Microbial Diseases of Osaka University was used and specimens were frozen within 1 hour after swab, while in our study specimens obtained from conjunctival swabs were immediately transferred to the Microbiology and Mycology Laboratory, where they were incubated aerobically in enriched Thioglycolate liquid medium.

The antibiotic resistance profile found in the current study raises significant concerns. The substantial proportion of eyes with methicillin-resistant and ceftazidime-resistant bacteria is especially concerning given that a combination of ceftazidime and vancomycin is one of the most widely used antibiotic regimens to treat endophthalmitis.[3,21] Vancomycin resistance was not found in our sample, showing that this medication remains a good choice for endophthalmitis treatment in the geographic region studied. Topical moxifloxacin is frequently used for endophthalmitis prophylaxis.[22] The presence of moxifloxacin-resistant bacteria in our study and in previous reports,[23] reinforces the importance of research investigating optimization of anti-sepsis before invasive ocular interventions.

There is a lack of a uniformly accepted recommendation regarding the exposure time and the number of drops of 5% PI before intraocular procedures, although some studies about dosage and concentration have been performed. [24,25] The present intervention was chosen based on its capacity of applying of a greater amount of PI than a single drop and because it results in a greater exposure time. Studies have shown that 1 or 2 drops of 5% PI to the ocular surface can significantly reduce bacterial colonization and the risk of endophthalmitis.[1,26,27] Our study demonstrated that a regimen with more frequent instillation (PI plus) started 30 minutes before intraocular intervention significantly reduced the bacterial load from the conjunctival flora. To the best of our knowledge, there is no study comparing the two different PI regimens employed in this study. However, there are some studies that indirectly corroborate and support the present study results.

Although the proportion of negative bacterial cultures on all tested media was significantly different before and after treatment in the PI plus group, the proportion of eyes with negative bacterial cultures on all tested media after PI administration was not different between both groups evaluated (univariate relative risk = 0.9345; 95% CI = 0.7916 to 1.103). This fact can be explained by an insufficient sensitivity of the univariate and of the multivariate models in detecting statistical differences probably because of a small sample size. This is an important limitation of this study. The absence of previous studies that compare similar regimens of PI instillation may have resulted in an underestimated sample size calculation.

The better antiseptic effect observed in the PI plus group compared to the PI group could be explained by a longer duration of PI exposure or a greater number of PI drops instilled. Hosseini et al. showed that longer exposure (15 minutes) to PI was more effective than shorter exposure (5 minutes) in preventing growth of bacterial isolates from patients diagnosed with postoperative endophthalmitis.[28] Moreover, Nentwich et al. concluded that copious use of PI in the preoperative period can result in a better irrigation of conjunctival crypts, resulting in a reduced risk of postoperative endophthalmitis.[29] Further study is warranted to investigate the relative importance of quantity of PI instilled versus duration of contact of the PI on the ocular surface.

One concern associated with PI eye drops is the potential for corneal toxicity.[30] Wille (1982) evaluated corneal swelling with pachymetry and endothelial cell loss with specular microscopy following cataract surgery and observed no increased postoperative corneal swelling when PI was used.[31] In the current study, there was no significant change in CCT after PI instillation in either group, but patient follow-up in the study was short. Of note, no patient demonstrated evidence of corneal toxicity during routine follow-up examinations in our department for at least two years after study completion.

Results of the current study indicate that instillation of three PI 5% eyedrops over 28 minutes significantly reduces the number of all negative bacterial cultures because of a greater number of PI drops instilled and/or because of the increased duration of exposure of the ocular surface to PI. This PI administration regimen may be more effective than instillation of a single eyedrop of PI 5%, although the relatively small sample size of the current study precludes definitive conclusions to be drawn. Further investigation is needed in order to optimize preoperative antisepsis with the ultimate goal of reducing the occurrence of postoperative endophthalmitis.

## Supporting information

S1 CONSORT checklist CONSORT 2010 checklist. Checklist of information to include when reporting a randomised trial.
(DOC)

S1 Data Sheet Excel file with data used for analysis. (XLSX)

S1 Original Protocol Original Research Protocol. (DOC)

S1 Protocol Translation Protocol methods section translation. English translation of the methods section extracted from the Original Research Protocol.
(DOC)

## References

[1] Speaker MG, Milch FA, Shah MK, Eisner W, Kreiswirth BN. Role of external bacterial flora in the pathogenesis of acute postoperative endophthalmitis. Ophthalmology. 1991;98: 639–49– discussion 650. 2062496 10.1016/s0161-6420(91)32239-5

[2] Bannerman TL, Rhoden DL, McAllister SK, Miller JM, Wilson LA. The source of coagulase-negative staphylococci in the Endophthalmitis Vitrectomy Study. A comparison of eyelid and intraocular isolates using pulsed-field gel electrophoresis. Arch Ophthalmol. 1997;115: 357–361. 9076208 10.1001/archopht.1997.01100150359008

[3] Schwartz SG, Flynn HW. Update on the prevention and treatment of endophthalmitis. Expert Rev Ophthalmol. 2014;9: 425–430. doi: 10.1586/17469899.2014.951331 26609317 10.1586/17469899.2014.951331 PMC4655603

[4] Moss JM, Sanislo SR, Ta CN. A prospective randomized evaluation of topical gatifloxacin on conjunctival flora in patients undergoing intravitreal injections. Ophthalmology. Elsevier; 2009;116: 1498–1501. doi: 10.1016/j.ophtha.2009.02.024 19501409 10.1016/j.ophtha.2009.02.024

[5] Ahmed Y, Scott IU, Pathengay A, Bawdekar A, Flynn HW. Povidone-iodine for endophthalmitis prophylaxis. Am J Ophthalmol. Elsevier; 2014;157: 503–504. doi: 10.1016/j.ajo.2013.12.001 24528933 10.1016/j.ajo.2013.12.001

[6] Modjtahedi BS, van Zyl T, Pandya HK, Leonard RE, Eliott D. Endophthalmitis After Intravitreal Injections in Patients With Self-reported Iodine Allergy. Am J Ophthalmol. Elsevier; 2016;170: 68–74. doi: 10.1016/j.ajo.2016.07.010 27448925 10.1016/j.ajo.2016.07.010

[7] Kıvanç SA, Kıvanç M, Bayramlar H. Microbiology of corneal wounds after cataract surgery: biofilm formation and antibiotic resistance patterns. J Wound Care. MA Healthcare London; 2016;25: 12–14–9. doi: 10.12968/jowc.2016.25.1.12 26762493 10.12968/jowc.2016.25.1.12

[8] Harun S, Srinivasan S, Hollingworth K, Batterbury M, Kaye S. Modification of classification of ocular chemical injuries. Br J Ophthalmol. BMJ Publishing Group Ltd; 2004;88: 1353–4– author reply 1354–5. doi: 10.1136/bjo.2004.046797/047308 15377570 10.1136/bjo.2004.046797/047308 PMC1772368

[9] Ferguson AW, Scott JA, McGavigan J, Elton RA, McLean J, Schmidt U, et al Comparison of 5% povidone-iodine solution against 1% povidone-iodine solution in preoperative cataract surgery antisepsis: a prospective randomised double blind study. Br J Ophthalmol. BMJ Group; 2003;87: 163–167. 12543744 10.1136/bjo.87.2.163 PMC1771501

[10] GERSHENFELD L. Povidone-iodine as a sporicide. Am J Pharm Sci Support Public Health. 1962;134: 78–81. 13898055

[11] LIANG K-Y ZEGER SL. Longitudinal data analysis using generalized linear models. Biometrika. Oxford University Press; 1986;73: 13–22. doi: 10.1093/biomet/73.1.13

[12] Liddell FD. Simplified exact analysis of case-referent studies: matched pairs; dichotomous exposure. J Epidemiol Community Health. BMJ Publishing Group Ltd; 1983;37: 82–84. doi: 10.1136/jech.37.1.82 10.1136/jech.37.1.82 PMC1052263 6875452

[13] Sakpal TV. Sample size estimation in clinical trial. Perspect Clin Res. 2010;1: 67–69. 21829786 PMC3148614

[14] Cimmino T, Le Page S, Raoult D, Rolain J-M. Contemporary challenges and opportunities in the diagnosis and outbreak detection of multidrug-resistant infectious disease. Expert Review of Molecular Diagnostics. Taylor & Francis; 2016;: 14737159.2016.1244005–13. doi: 10.1080/14737159.2016.1244005 27690721 10.1080/14737159.2016.1244005

[15] Bhat SS, Undrakonda V, Mukhopadhyay C, Parmar PV. Outbreak of multidrug-resistant acute postoperative endophthalmitis due to Enterobacter aerogenes. Ocul Immunol Inflamm. 2014;22: 121–126. doi: 10.3109/09273948.2013.830752 24063692 10.3109/09273948.2013.830752

[16] Deng C, Zhang W, Yuan Y, Yao A, Hu Y, Yu F, et al Report: Prevention infection of newborn nosocomial and distribution of multiple drug resistant organism of the medicinal. Pak J Pharm Sci. 2016;29: 361–365. 27005503

[17] Tsitsopoulos PP, Iosifidis E, Antachopoulos C, Anestis DM, Karantani E, Karyoti A, et al Nosocomial bloodstream infections in neurosurgery: a 10-year analysis in a center with high antimicrobial drug-resistance prevalence. Acta Neurochir. Springer Vienna; 2016;158: 1647–1654. doi: 10.1007/s00701-016-2890-5 27452903 10.1007/s00701-016-2890-5

[18] Nentwich MM, Kollmann KHM, Meshack J, Ilako DR, Schaller UC. Microbial contamination of multi-use ophthalmic solutions in Kenya. Br J Ophthalmol. BMJ Publishing Group Ltd; 2007;91: 1265–1268. doi: 10.1136/bjo.2007.116897 17475714 10.1136/bjo.2007.116897 PMC2000994

[19] Mastropasqua L. Collagen cross-linking: when and how? A review of the state of the art of the technique and new perspectives. Eye and Vis. JAMA …; 2015;2: 842 doi: 10.1186/s40662-015-0030-6 26665102 10.1186/s40662-015-0030-6 PMC4675057

[20] Inoue Y, Usui M, Ohashi Y, Shiota H, Yamazaki T, Preoperative Disinfection Study Group. Preoperative disinfection of the conjunctival sac with antibiotics and iodine compounds: a prospective randomized multicenter study. Jpn J Ophthalmol. Springer Japan; 2008;52: 151–161. doi: 10.1007/s10384-008-0517-y 18661264 10.1007/s10384-008-0517-y

[21] Cornut P-L, Chiquet C. [Intravitreal injection of antibiotics in endophthalmitis]. J Fr Ophtalmol. 2008;31: 815–823. 19107050 10.1016/s0181-5512(08)74405-x

[22] Rudnisky CJ, Wan D, Weis E. Antibiotic choice for the prophylaxis of post-cataract extraction endophthalmitis. Ophthalmology. Elsevier; 2014;121: 835–841. doi: 10.1016/j.ophtha.2013.08.046 24326107 10.1016/j.ophtha.2013.08.046

[23] Miller D, Flynn PM, Scott IU, Alfonso EC, Flynn HW. In vitro fluoroquinolone resistance in staphylococcal endophthalmitis isolates. Arch Ophthalmol. American Medical Association; 2006;124: 479–483. doi: 10.1001/archopht.124.4.479 16606872 10.1001/archopht.124.4.479

[24] de Kaspar HM, Chang RT, Singh K, Egbert PR, Blumenkranz MS, Ta CN. Prospective Randomized Comparison of 2 Different Methods of 5% Povidone-Iodine Applications for Anterior Segment Intraocular Surgery. Arch Ophthalmol. American Medical Association; 2005;123: 161–165. doi: 10.1001/archopht.123.2.161 15710810 10.1001/archopht.123.2.161

[25] Silas MR, Schroeder RM, Thomson RB, Myers WG. Optimizing the antisepsis protocol: Effectiveness of 3 povidone–iodine 1.0% applications versus a single application of povidone–iodine 5.0%. J Cataract Refract Surg. 2017;43: 400–404. doi: 10.1016/j.jcrs.2017.01.007 28410725 10.1016/j.jcrs.2017.01.007

[26] Apt L, Isenberg S, Yoshimori R, Paez JH. Chemical preparation of the eye in ophthalmic surgery. III. Effect of povidone-iodine on the conjunctiva. Arch Ophthalmol. 1984;102: 728–729. 6721765 10.1001/archopht.1984.01040030584025

[27] Isenberg SJ, Apt L, Yoshimori R, Khwarg S. Chemical preparation of the eye in ophthalmic surgery. IV. Comparison of povidone-iodine on the conjunctiva with a prophylactic antibiotic. Arch Ophthalmol. 1985;103: 1340–1342. 2994609 10.1001/archopht.1985.01050090092039

[28] Hosseini H, Ashraf MJ, Saleh M, Nowroozzadeh MH, Nowroozizadeh B, Abtahi MB, et al Effect of povidone-iodine concentration and exposure time on bacteria isolated from endophthalmitis cases. J Cataract Refract Surg. Elsevier; 2012;38: 92–96. doi: 10.1016/j.jcrs.2011.06.030 21983301 10.1016/j.jcrs.2011.06.030

[29] Nentwich MM, Ta CN, Kreutzer TC, Li B, Schwarzbach F, Yactayo-Miranda YM, et al Incidence of postoperative endophthalmitis from 1990 to 2009 using povidone-iodine but no intracameral antibiotics at a single academic institution. J Cataract Refract Surg. Elsevier; 2015;41: 58–66. doi: 10.1016/j.jcrs.2014.04.040 25532634 10.1016/j.jcrs.2014.04.040

[30] Mac Rae SM, Brown B, Edelhauser HF. The corneal toxicity of presurgical skin antiseptics. Am J Ophthalmol. 1984;97: 221–232. 6696033 10.1016/s0002-9394(14)76094-5

[31] Wille H. Assessment of possible toxic effects of polyvinylpyrrolidone-iodine upon the human eye in conjunction with cataract extraction. An endothelial specular microscope study. Acta Ophthalmol (Copenh). 1982;60: 955–960. 7170937 10.1111/j.1755-3768.1982.tb00627.x

