# Supplementary material for: Comparative efficacy of two different topical povidone-iodine 5% regimens in reducing conjunctival bacterial flora: A randomized parallel double-masked clinical trial
Source: PLoS One. 2017 Dec 19;12(12):e0189206. doi: 10.1371/journal.pone.0189206 (PMC5736199; doi:10.1371/journal.pone.0189206)
Supplement: S1 Original Protocol — (DOC) [file pone.0189206.s003.doc]

**S1 Original Protocol. Original research protocol.**

Estudo comparativo da eficácia de dois regimes de instilação do colírio de iodo-povidona a 5% em reduzir a flora microbiana conjuntival

Comparative study of the efficacy of two different regimens of instillation of povidone-iodine 5% eye drops in reducing conjunctival bacterial flora

27 de maio de 2010

Prof. Dr. Rodrigo Jorge

FMRP-USP

1. Resumo:

A endoftalmite pós-operatória, embora rara, é uma das mais temidas complicações da cirurgia intra-ocular podendo levar a cegueira Diversos estudos foram realizados visando definir quais são as condutas mais eficazes para minimizar os riscos de infecção. Como as bactérias encontradas nos casos de endoftalmite são freqüentemente as mesmas espécies encontradas na pálpebra e conjuntiva do próprio paciente, os novos estudos buscam reduzir a flora conjuntival bacteriana naqueles que serão submetidos a cirurgias, com o objetivo de diminuir o risco de endoftalmite.

O método que comprovadamente reduz a flora bacteriana e a chance do indivíduo evoluir com infecção intra-ocular pós-operatória é o colírio de iodo-povidona (PVPI) pré-operatório. Embora, muitos estudos tenham sido feitos tentando comparar o colírio de PVPI 5% com demais colírios antibióticos e associações, há poucos estudos que comparam a eficácia do colírio de PVPI 5% utilizando diferentes posologias, visando otimizar seu efeito.

Dessa forma, é proposta a presente pesquisa para investigar diferentes posologias com relação ao uso do colírio de PVPI% com o intuito de melhor definir sua eficácia na redução da flora conjuntival e no conseqüente combate profilático da endoftalmite exógena.

2. Introdução/Justificativa:

2.1 Endoftalmite:

Endoftalmite é uma inflamação intra-ocular potencialmente grave que pode ocorrer por disseminação hematogênica, a partir de um local remoto, ou ocorrer por meio de uma complicação grave de cirurgia intra-ocular ou por trauma ocular. Endoftalmite não é, necessariamente, um processo infeccioso destarte, uma grave inflamação não-infecciosa pós-operatória denomina-se endoftalmite estéril. Endoftalmite infecciosa ocorre a partir de infecção das estruturas internas do olho por bactérias, vírus, fungos e protozoários, constituindo uma devastadora complicação em cirurgia intra-ocular.

Os pacientes que apresentam endoftalmite pós-operatória cursam, freqüentemente, com perda visual progressiva, edema palpebral, hiperemia conjuntival, hipópio e dor ocular. A acuidade visual pode deteriorar a percepção de luz e progredir para perda total da visão nos casos mais graves.

A incidência de endoftalmite no pós-operatório da cirurgia de catarata e glaucoma encontra-se entre 0.08% e 0.12%, respectivamente. (Speaker et al., 1998; Wu et al., 2006; Aaberg Jr et al., 1998).  A injeção intra-vítrea também tem um reconhecido risco de endoftalmite, embora a aplicação intra-vítrea de antiangiogênicos como o bevacizumabe, por exemplo, demonstrem uma taxa muito baixa de endoftalmite em torno de 0.019%. A injeção intra-vítrea de corticosteróide, como o acetato de triancinolona, apresenta uma maior incidência de endoftalmite, estimada em 0.2% por injeção, podendo atingir índices até maiores, como sugerem Moshfeghi et al. (2003), cuja incidência de endoftalmite em culturas bacterianas positivas após injeção intra-vítrea de triancinolona foi maior (0.87%) do que por outros procedimentos intra-oculares relatados (Sakamoto et al., 2004; Trinavarat et al., 2006; AC Westfall et al., 2005; Jager et al., 2004; Mason et al., 2008.

Estudos têm demonstrado que a flora bacteriana externa do paciente é, na maioria dos casos, a própria fonte de infecção. Speaker et al. (1991a) verificaram que 82% dos casos de endoftalmite, cujos microrganismos foram isolados do vítreo, os mesmos foram geneticamente indistinguíveis dos isolados após coleta de material da pálpebra do paciente, conjuntiva, ou nariz.

O fato de endoftalmite ocorrer em um número tão pequeno de casos não está ainda bem esclarecido. A resposta a esta pergunta pode ser encontrada na importância de outros fatores como a virulência da cepa, a redução temporária ou permanente na imunidade do paciente, a densidade de colonização bacteriana dos tecidos do olho e do tamanho do inóculo (Walker et al., 1986; González et al., 2001).

O objetivo da anti-sepsia é eliminar ou reduzir significativamente o número de microorganismos no campo cirúrgico no momento da cirurgia. As pálpebras e a conjuntiva são consideradas fontes bastante comuns de bactérias, podendo levar a endoftalmite. (Bannerman et al.,1997) Desta forma, acredita-se que se reduzindo o número e o crescimento de bactérias na superfície e anexos oculares previamente à cirurgia, o risco de infecção pós-operatória seria menor.

Com este fim, várias medidas profiláticas foram relatadas e têm sido usadas. Entre elas, o uso de iodo-povidona (PVPI) no pré-operatório, uso de antibióticos subconjuntival no intra-operatório e isolamento de cílios, irrigação salina pré-operatória. Ciulla et al. (2002), em revisão sistemática da literatura, demonstraram que o uso de PVPI no preparo pré-operatório das cirurgias intra-oculares foi a única medida considerada comprovadamente capaz de diminuir a incidência de endoftalmite pós-facectomia.

2.2 Polivinilpirrolidona:

O iodo, isoladamente, tem seu uso limitado na profilaxia antibacteriana pré-operatória em humanos pela baixa solubilidade em água e pela elevada toxicidade. O uso, em larga escala, deste antimicrobiano tornou-se viável a partir do desenvolvimento dos iodoforos, pela ligação do iodo à macromolécula de polivinilpirrolidona (povidona).

Polivinilpirrolidona (PVP; E.U. Pharmacopeia (USP) nome, povidona) é o homopolímero de N-vinil-2-pirrolidona. Embora PVP seja considerado um polímero não-iônico, tem propriedades de desintoxicação que são de grande interesse nos cuidados de saúde.

Algumas propriedades do iodo são alteradas e aprimoradas pela combinação do iodo com uma molécula carreadora, como a povidona. Estas soluções de iodo são chamadas iodophors (iodo; phor=carreadora). Os iodoforos apresentam melhor atividade antibacteriana e estabilidade, menos toxicidade, reduzida pressão de vapor, menos odor e aumentaram a solubilidade em água, facilitando a diluição (Gershenfeld et al., 1957; Shelanski et al., 1956). Complexo iodado, como o iodo-povidona, reduz sua toxicidade dez vezes e ainda permite que a ação antibacteriana possa ocorrer. Isso ocorre através da formação de íons de iodo por agentes redutores no polímero, que, em seguida, esses complexos com iodo molecular geram mais íons de iodo.

Complexos de PVP também podem ser feitos com mercúrio, a nicotina, cianeto e outros materiais tóxicos para reduzir seus efeitos danosos. PVP tem muito baixa toxicidade sistêmica, não apresenta características de rejeição imunológica, e é facilmente excretada pelos rins, com um peso molecular de até 100.000 d (Ocular Pharmacology Clinical, 2001).

Maumenee et al. (1951) apreciaram os possíveis efeitos do iodo no olho em 1951, quando uma redução na flora ocular foi relatada após a aplicação de solução de iodo para a pele. Iodóforos foram notificados para reduzir a flora da pele ao redor dos olhos, em 1970 (Chase et al., 1970), e só mais tarde a combinação específica de iodo-povidona foi formulada para uso oftálmico diretamente.

Estudos demonstram que Iodo-povidona 5% é seguro e eficaz na redução do número de bactérias na superfície ocular no momento da cirurgia. Apt et al. (1984) verificaram que 1 a 2 gotas de iodo-povidona a 5% colocada no olho reduziu o número de bactérias em 91%. Este estudo de 1984 foi o primeiro ensaio controlado usando a iodo-povidona como solução oftálmica.

Um estudo realizado em Nova York com 8000 pacientes submetidos à cirurgia de catarata demonstrou que o uso da solução de iodo-povidona colocada no olho, antes da cirurgia, reduziu a taxa de endoftalmite de 0,24% para 0,06% (p <0,03) (Speaker et al., 1991). Iodo-povidona provou ser um anti-séptico seguro para a pele periocular e conjuntiva, sendo eficaz contra vírus, bactérias, rickéttsias, fungos, protozoários e esporos. Ele rompe as membranas celulares em contato, matando os microorganismos, age rapidamente, e pode ser instilado imediatamente antes da cirurgia. Speaker & Menikoff. (1991) analisaram o uso do colírio de iodo-povidona 5% na profilaxia de infecção pós-operatória e encontraram uma incidência significativamente menor de endoftalmite cultura-positiva (P <0,03) em relação ao uso de solução de proteína de prata. Eles argumentaram que a redução da flora da superfície ocular é provavelmente o melhor método na prevenção de endoftalmite.

Dereklis et al. (1994) mostraram redução de culturas positivas da conjuntiva, de 66% para 30% dos olhos estudados, após instilar uma gota de 5% iodo-povidona.

Soluções de iodo-povidona sem detergente mesmo em concentrações maiores, como o colírio de PVPI a 10%, provocam toxicidade corneana mínima enquanto que os outros anti-sépticos de pele pré-cirúrgicos testados são tóxicos para a córnea. A instilação de colírio de PVPI a 10% no saco conjuntival causou alguma inflamação da superfície externa do olho enquanto que a diluição a 5% não apresentou essa inflamação e ainda teve boa atividade antimicrobiana, como evidenciada pela redução significativa da flora bacteriana na conjuntiva, apresentando assim baixa toxicidade e alta eficácia. (Mac Rae et al., 1984; Apt et al., 1989).

PVPI ISOLADO VERSUS PVPI E ASSOCIAÇÕES:

Autores descrevem que a combinação de PVPI e antibióticos tópicos instilados no pré-operatório resultam em maior diminuição da flora conjuntival do que a encontrada com o uso isolado desta substância. (Isenberg et al., 1985; Kaspar et al., 2004).

Todavia, existem questionamentos e incertezas em relação à forma de uso desses antibióticos e quais deles seriam os mais adequados na profilaxia pré-operatória, uma vez que muitos antibióticos previamente estudados (ciprofloxacino, norfloxacino, tobramicina, neomicina) apresentam susceptibilidade bacteriana sujeita a mudanças com o decorrer dos anos, e como conseqüências disso, muitas bactérias já apresentam altos níveis de resistência. (Hodge et al., 1995; Seppala et al., 2004).

Fluoroquinolonas de quarta geração têm taxas mais baixas de resistência bacteriana que ofloxacino e ciprofloxacino, embora não estejam livres do desenvolvimento de resistência bacteriana. (Mah et al., 2004).

Embora pesquisas revelem que a maioria dos cirurgiões de catarata prescreve antibióticos tópicos no pré-operatório, existem várias maneiras descritas para sua utilização, desde a utilização por dias antes da cirurgia até nas horas precedentes ao procedimento operatório (Ta et al., 2003; Isenberg et al., 1985; Sousa et al.,. 2003).

Arantes e al. (2008) compararam a eficácia entre os colírios de Ciprofloxacino e Gatifloxacino na redução da flora bacteriana conjuntival no pré-operatório. O estudo averiguou uma diminuição do número de culturas conjuntivais positivas com o uso de colírios de ciprofloxacino e gatifloxacino, aplicados uma hora antes da cirurgia, sendo esta redução significante com o gatifloxacino. Contudo, após a aplicação do PVPI, foram encontrados os menores números de culturas positivas e não houve diferença entre os grupos que utilizaram ciprofloxacino e gatifloxacino no pré-operatório.

Moss et al.(2009) demonstraram que aplicação de gatifloxacina tópica, por 3 dias, reduz significativamente a porcentagem de olhos com culturas bacterianas positivas em comparação aos olhos não tratados. No entanto, após a aplicação do PVPI, nos olhos não tratados (controle) e nos olhos pré-tratados com gatifloxacina tópica experimentaram reduções drásticas no número de bactérias positivas culturas a um nível que não foi significativamente diferente entre os 2 grupos. Estes resultados sugerem que o uso por 3 dias de gatifloxacina tópica em combinação com o PVI não ofereceu mais redução da contaminação bacteriana do que o PVI sozinho.

Justificativa do estudo:

A endoftalmite pós-operatória é uma das mais temidas complicações da cirurgia intra-ocular, sendo o uso isolado do colírio de PVPI a 5% comprovadamente eficaz na profilaxia. Diferentes metodologias na aplicação do colírio de PVPI foram pouco estudadas para definir qual é o protocolo mais eficaz na aplicação do PVPI em reduzir a flora conjuntival bacteriana. Nosso estudo visa avaliar a eficácia do colírio de PVPI a 5% em reduzir a flora bacteriana em fundo de saco conjuntival com a aplicação de 3 gotas do colírio nos tempos: 00min, 20min e 28min (tempo contado a partir da aplicação da primeira gota de PVPI) em comparação ao procedimento padrão, que corresponde à aplicação de apenas 1 gota do colírio de PVPI 2min antes de qualquer procedimento cirúrgico intra-ocular.

Neste estudo, pretendemos verificar se o novo protocolo de profilaxia de endoftalmite pós-cirúrgica apresentará melhor eficácia na redução da flora conjuntival bacteriana em relação ao tratamento padrão, comparando a eficácia dos dois métodos em eliminar ou reduzir o número de microorganismos presentes no fundo de saco conjuntival.

3. Objetivos:

Geral

- Avaliar a eficácia da aplicação de 3 gotas do colírio de PVPI a 5% na redução da flora bacteriana no fundo de saco conjuntival para a prevenção de endoftalmite pos-operatória.

Específicos

- Avaliar a eficácia da aplicação do colírio de PVPI 5% na redução quantitativa e qualitativa da flora bacteriana conjuntival nos diferentes protocolos.

- Quantificar o número de colônias nas amostras em que houve crescimento bacteriano.

- Identificar que bactérias cresceram nos meios de cultura estudados.

- Verificar os efeitos do procedimento sobre a espessura corneana pela paquimetria de não contato.

Riscos e Benefícios:

Os pacientes, ao serem incluídos na pesquisa, não apresentarão riscos decorrentes de complicações pós-operatórias, uma vez que não será realizado nenhum procedimento cirúrgico adicional, sendo selecionados os pacientes acompanhados no ambulatório do serviço de Retina e Vitreo do Hospital das Clínicas da FMRP_USP.

Os pacientes em estudo podem apresentar reação alérgica ao colírio, mesmo tendo história pessoal negativa de alergia ao iodo. Há possibilidade de os pacientes apresentarem desepitelização corneana e quemose conjuntival após uso do colírio, sendo um processo reversível em 1-3 dias e de ocorrência rara na concentração de 5%.

Os pacientes submetidos à análise de material conjuntival, pelo laboratório de Microbiologia, terão a vantagem de terem seu tratamento melhor direcionado pelo médico que o acompanha em caso de eventual endoftalmite uma vez que se poderá saber que micro-organismo faz parte de sua flora conjuntival. Muitos deles realizarão cirurgias oftalmológicas (como cirurgia de catarata e/ou vitrectomia posterior) e poderão se beneficiar das informações relativas à flora conjuntival dos mesmos, com relação à profilaxia de endoftalmite. Alguns pacientes realizam injeções intravítreas de repetição (a cada 6/8 semanas) e também podem se beneficiar das informações obtidas no estudo. Caso o esquema com 3 gotas de PVPI se mostre mais eficiente, o mesmo passará a ser aplicado e beneficiará os pacientes da pesquisa, assim como os demais pacientes do HCFMRP-USP.

4. Materiais e Métodos:

Desenho do estudo:

Estudo prospectivo e randomizado.

Seleção de pacientes:

Serão incluídos no estudo 40 pacientes atendidos pelo serviço de Retina e Vítreo do Hospital das Clínicas da Faculdade de Medicina de Ribeirão Preto agendados para procedimentos cirúrgicos, de forma aleatória, desde que preencham os critérios de inclusão e respeitem os critérios de exclusão.

Critérios de inclusão:

- Idade: acima de 18 anos de idade;

- Ausência de infecção sistêmica ou infecção ocular durante o período do estudo.

- ausência de doenças auto-imune ou de terapia imunossupressora.

- sem uso de antibiótico dentro dos últimos 10 dias que precedem o procedimento.

- ausência de cirurgia ocular ou trauma no olho que será envolvido, nos últimos 30 dias.

- sem história de alergia a iodo e seus derivados.

- Termo de consentimento livre e esclarecido por escrito devidamente assinado (TCLE).

Critérios de exclusão:

- Cirurgia intra-ocular nos últimos 30 dias;

- Uso de antibiótico nos últimos 10 dias que precedem o procedimento.

- Tratamento com radiação ionizante na região da face, crânio e pescoço;

- Diagnóstico de Diabetes Mellitus

- Infecção sistêmica, infecção ocular, blefarite, ectrópio, entrópio ou distriquíase.

- Uso conhecido abusivo de álcool ou drogas;

- Condições médicas ou psicológicas que impeçam o paciente de concluir o estudo ou assinar o consentimento informado;

- Doença significativa e não controlada que, na opinião do investigador, possa excluir o paciente do estudo;

- Impedimento ou capacidade legal limitada;

- Participação em outro estudo clínico nos últimos 30 dias

Procedimentos do Estudo:

Os pacientes aptos para admissão, após serem esclarecidos e terem assinado o termo de consentimento, serão incluído no estudo sendo distribuídos aleatoriamente em dois grupos por meio de sorteio.

No Grupo 1, que será chamado de grupo PVPI, os pacientes receberão 3 gotas de PVPI 5% no olho estudado. No Grupo 2, que será chamado Grupo Controle, os pacientes selecionados receberão apenas 1 gota do colírio de PVPI 5%.

No Grupo PVPI, cada paciente, inicialmente será submetido a uma paquimetria antes da aplicação do colírio de PVPI 5%.

Realizar-se-á uma paquimetria inicial e avaliação biomicroscópica do segmento anterior, em seguida, pingar-se-á 1 gota de colírio anestésico de Cloridrato de Proximetacaína a 0,5% e colher-se-á uma amostra do material em fundo de saco conjuntival com swab estéril 5 minutos antes da aplicação da 1ª. gota de PVPI colírio. A aplicação da 1ª gota PVPI 5% será feita no tempo 00min. Aplicar-se-á a 2ª. gota de PVPI no tempo 20min e a 3ª gota no tempo 28min. Dois minutos após pingada a 3ª. gota de PVPI, ou seja, no tempo 30min, colher-se-á uma 2ª. amostra do fundo de saco conjuntival.

Finalmente, após a coleta da 2ª. amostra do fundo de saco conjuntival, o paciente realizará uma segunda paquimetria e nova avaliação biomicroscópica do segmento anterior pela lâmpada de fenda para seguimento e avaliação de algum possível dano ou efeito adverso.

No Grupo Controle, os pacientes serão submetidos ao mesmo procedimento descrito acima, exceto pela substituição da 1ª e 2ª gotas de PVPI 5% por Solução Fisiológica 0,9% (SF0,9%) com finalidade de servir como grupo comparativo do estudo.

Imediatamente antes de cada gota de PVPI ou de SF0,9% é aplicado 1 gota de colírio anestésico de Cloridrato de Proximetacaína a 0,5%.

As amostras serão transferidas imediatamente para o laboratório de microbiologia, onde serão incubados em meio líquido de tioglicolato (caldo de carne, a 37 ° C) e três meios de cultura sólidos (ágar chocolate; Trypcase Soy Agar com sangue de carneiro 5% e Ágar Sabouraud a 37 ° C). O meio líquido deve ser repicado no momento em que aparece turvo ou após 5 dias de incubação sem turvação para melhorar a sensibilidade do processo.

FLUXOGRAMA

Grupo PVPI e Grupo Controle


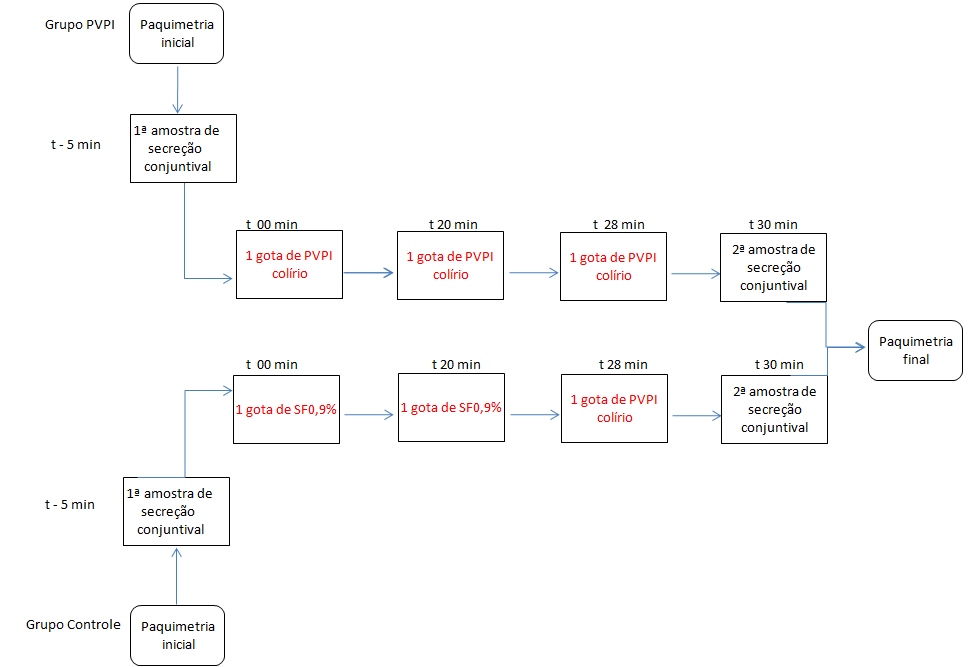


Métodos estatísticos:

A comparação pré x pós-tratamento será realizada por Análise de Variância, dependendo da distribuição apresentada pelas variáveis analisadas. Adotaremos o nível de significância estatística 0.05.

5. Referências:

Aaberg TM Jr, Flynn HW Jr, Schiffman J, Newton J. Nosocomial acute-onset postoperative endophthalmitis survey; a 10-year review of incidence and outcomes. Ophthalmology 1998;105:1004–1010.

Apt L, Isenberg S, Yoshimori R, Paez JH. Chemical preparation of the eye in ophthalmic surgery. III: effect of povidone-iodine on the conjunctiva. Arch Ophthalmol 1984; 102:728–729.

Apt, L., Isenberg, S.J., Yoshimori, R., et al. Outpatient topical use of povidone–iodine in preparing the eye for surgery. Ophthalmology. 1989; 96:289–292.

Arantes, Tiago Eugênio Faria e et al. Flora bacteriana conjuntival após uso tópico de ciprofloxacino e gatifloxacino em cirurgia de catarata. Arq. Bras. Oftalmol. [online]. 2008, vol.71, n.2, pp. 191-196).

Bannerman TL, Rhoden DL, McAllister SK, et al. The source of coagulase-negative staphylococci in the Endophthalmitis Vitrectomy Study; a comparison of eyelid and intraocular isolates using pulsed-field gel electrophoresis; the Endophthalmitis Vitrectomy Study Group. Arch Ophthalmol 1997; 115:357–361.

Caldwell DR, Kastl PR, Cook J, Simon J. Povidone-iodine: it is efficacy as a preoperative conjunctival and periocular preparation. Ann Ophthalmol 1984; 16:577, 580.

Chase R C, Ellis P P. Iodophors and skin asepsis: Iodophors as skin antiseptics before ophthalmic surgery. Ann Ophthalmol. 1970;12:312–317.

Clinical ocular pharmacology. Jimmy D Bartlett and Siret D Jaanus.. Butterworth-Heinemann, 4th edition, 2001, p-33).

Ciulla TA, Starr MB, Masket S. Bacterial endophthalmitis prophylaxis for cataract surgery. An evidence-based update. Ophthalmology 2002;109:13–26.

Dereklis DL, Bufidis TA, Tsiakiu EP, Palassopoulos SI: Preoperative ocular disinfection by the use of povidone-iodine 5%. Arc Ophthalmol 1994; 72:627-630

Dickey JB, Thompson KD, Jay WM. Anterior chamber aspirate cultures after uncomplicated cataract surgery. Am J Ophthalmol. 1991;112(3):278-82. Comment in: Am J Ophthalmol. 1992;113(2):221-2.

Gershenfeld L. Povidone-iodine as a topical antiseptic. Am J Surg 1957; 94:938-9.

González Bandrés C, Carrilero Ferrer MJ, Buznego Suárez L, García Claramunt MA, Méndez Llata M, Paredes B, Moriche Carretero M. [Efficacy of topical povidone-iodine applied the day before cataract surgery to reduce conjunctival flora](http://www.ncbi.nlm.nih.gov/pubmed/11340513?ordinalpos=99&itool=EntrezSystem2.PEntrez.Pubmed.Pubmed_ResultsPanel.Pubmed_DefaultReportPanel.Pubmed_RVDocSum). Arch Soc Esp Oftalmol. 2001 Apr;76(4):229-34. Spanish.

Han, D.P., Wisniewski, S.R., Wilson, L.A., et al. Spectrum and susceptibilities of microbiologic isolates in the Endophthalmitis Vitrectomy Study. Am. J. Ophthalmol.122:1–17, 1996.

Hodge W, Biu D. Frequency of recovery of ciprofloxacin-resistant ocular isolates following topical cirpofloxacin. Invest Ophthalmol Vis Sci 1995;36:155.

Isenberg S, Apt L, Yoshimori R, Khwarg S. Chemical preparation of the eye in ophthalmic surgery. IV. Comparison of povidone-iodine on the conjuntiva with a prophylactic antibiotic. Arch Ophthalmol. 1985;103(9):1340-2.

Jager RD, Aiello LP, Patel SC, Cunningham ET Jr. Risks of intravitreous injection: a comprehensive review. Retina 2004; 24: 676-698.

Kaspar HM, Chang RT, Singh K, et al. Prospective randomized comparison of 2 different methods of 5% povidone- iodine applications for anterior segment intraocular surgery. Arch Ophthalmol 2005; 123:161–165

Kaspar HM, Chang RT, Shriver EM, et al. Three-day application of topical ofloxacin reduces the contamination rate of microsurgical knives in cataract surgery. A prospective randomized study. Ophthalmology 2004; 111:1352–1355.

[Mac Rae SM](http://www.ncbi.nlm.nih.gov/pubmed?term=), [Brown B](http://www.ncbi.nlm.nih.gov/pubmed?term=), [Edelhauser HF](http://www.ncbi.nlm.nih.gov/pubmed?term=). The corneal toxicity of presurgical skin antiseptics. Am J Ophthalmol. 1984 Feb;97(2):221-32.

Mah FS. Fourth-generation fluoroquinolones: new topical agents in the war on ocular bacterial infections. Curr Opin Ophthalmol 2004;15:316-20.

Mason JO 3rd, White MF, Feist RM, Thomley ML, Albert MA, Persaud TO, Yunker JJ, Vail RS. [Incidence of acute onset endophthalmitis following intravitreal bevacizumab (Avastin) injection.](http://www.ncbi.nlm.nih.gov/pubmed/18398358?ordinalpos=15&itool=EntrezSystem2.PEntrez.Pubmed.Pubmed_ResultsPanel.Pubmed_DefaultReportPanel.Pubmed_RVDocSum) Retina. 2008 Apr;28(4):564-7.

Maumenee A E, Michler R C. Sterility of the operative field after ocular surgery. Pac Coast Oto-Ophthalmol Soc. 1951;32:172–183

Moshfeghi DM, Kaiser PK, Scott IU, et al. Acute endophthalmitis following intravitreal triamcinolone acetonide injection. Am J Ophthalmol. 2003;136:791-796.

# [**Moss JM**](http://www.ncbi.nlm.nih.gov/pubmed?term=), [**Sanislo SR**](http://www.ncbi.nlm.nih.gov/pubmed?term=), [**Ta CN**](http://www.ncbi.nlm.nih.gov/pubmed?term=). A prospective randomized evaluation of topical gatifloxacin on conjunctival flora in patients undergoing intravitreal injections. **Ophthalmology.** 2009 Aug;116(8):1498-501.

Sakamoto T, Enaida H, Kubota T, et al. Incidence of acute endophthalmitis after triamcinolone-assisted pars plana vitrectomy. Am J Ophthalmol. 2004;138:137-138.

Samad A, Solomon LD, Miller MA, Mendelson J. Anterior chamber contamination after uncomplicated phacoemulsification and intraocular lens implantation. Am J Ophthalmol. 1995;120(2):143-50.

Seppala H, Al-Juhaish M, Jarvinen H, et al. Effect of prophylactic antibiotics on

antimicrobial resistance of viridans streptococci in the normal flora of cataract surgery patients. J Cataract Refract Surg 2004;30:307-15.

Shelanski HÁ, Shelanski MV. PVP-iodine: history, toxicity and therapeutic uses. J Int Coll Surg 1956;25:727-34.

Sousa LB. Prevenção da infecção na cirurgia intra-ocular. In: Sousa LB, Freitas D, Höfling-Lima AL, Nishiwaki-Dantas MC, editores, Manual de prevenção da infecção nos procedimentos oftalmológicos. São Paulo: Lemos; 2003. p.129-34

[Speaker MG](http://www.ncbi.nlm.nih.gov/pubmed?term=), [Milch FA](http://www.ncbi.nlm.nih.gov/pubmed?term=), [Shah MK](http://www.ncbi.nlm.nih.gov/pubmed?term=), [Eisner W](http://www.ncbi.nlm.nih.gov/pubmed?term=), [Kreiswirth BN](http://www.ncbi.nlm.nih.gov/pubmed?term=). Role of external bacterial flora in the pathogenesis of acute postoperative endophthalmitis. Ophthalmology. 1991 May;98(5):639-49; discussion 650.

Speaker MG, Menikoff JA: Prophylaxis of endophthalmitis with topical povidone-iodine. Ophthalmol 98:1769-1775, 1991

Speaker MG, Milch FA, Shah MK, Eisner W, et al: Role of external bacterial flora in the pathogenesis of acute postoperative endophthalmitis survey. A 10-year review of incidence and outcomes. Ophthalmol 105:1004-1010, 1998

Ta CN, Chang RT, Singh K, Egbert PR, Shriver EM, Blumenkranz MS, et al. Antibiotic resistance patterns of ocular bacterial flora. A prospective study of patients undergoing anterior segment surgery. Ophthalmology. 2003; 110(10):1946-51.

Ta CN,  Singh K,  Egbert PR,  Kaspar HM. Prospective comparative evaluation of povidone–iodine (10% for 5 minutes versus 5% for 1 minute) as prophylaxis for ophthalmic surgery. Journal of Cataract & Refractive Surgery, Volume 34, Issue 1, January 2008, Pages 171-172

[Trinavarat A](http://www.ncbi.nlm.nih.gov/pubmed?term=), [Atchaneeyasakul LO](http://www.ncbi.nlm.nih.gov/pubmed?term=), [Nopmaneejumruslers C](http://www.ncbi.nlm.nih.gov/pubmed?term=), [Inson K](http://www.ncbi.nlm.nih.gov/pubmed?term=)**. Reduction of endophthalmitis rate after cataract surgery with preoperative 5% povidone-iodine. Dermatology**, Jan 2006; 212 Suppl 1: 35-40.

Walker CB, Claone CM. Incidence of conjunctival colonization by bacteria capable of causing postoperative endophthalmitis. J R Soc Med 1986; 79: 520-521.

Westfall AC, Osborn A, Kuhl D, Benz MS, Mieler WF, Holz ER. **Acute endophthalmitis incidence: intravitreal triamcinolone. Arch Ophthalmol**, Aug 2005; 123: 1075-7

Wu PC, Li M, Chang SJ, Teng MC, Yow SG, Shin SJ, Kuo HK. [Risk of endophthalmitis after cataract surgery using different protocols for povidone- iodine preoperative disinfection.](http://www.ncbi.nlm.nih.gov/pubmed/16503776?ordinalpos=49&itool=EntrezSystem2.PEntrez.Pubmed.Pubmed_ResultsPanel.Pubmed_DefaultReportPanel.Pubmed_RVDocSum). J Ocul Pharmacol Ther. 2006 Feb;22(1):54-61.
