# Supplementary material for: Comparative efficacy of two different topical povidone-iodine 5% regimens in reducing conjunctival bacterial flora: A randomized parallel double-masked clinical trial
Source: PLoS One. 2017 Dec 19;12(12):e0189206. doi: 10.1371/journal.pone.0189206 (PMC5736199; doi:10.1371/journal.pone.0189206)
Supplement: S1 Protocol Translation — English translation of the methods section extracted from the Original Research Protocol. (DOC) [file pone.0189206.s004.doc]

**S1 Protocol translation. Protocol methods section translation.** English translation of the methods section extracted from the Original Research Protocol.

Estudo comparativo da eficácia de dois regimes de instilação do colírio de iodo-povidona a 5% em reduzir a flora microbiana conjuntival

Comparative study of the efficacy of two different regimens of instillation of povidone-iodine 5% eye drops in reducing conjunctival bacterial flora

27 de maio de 2010

Prof. Dr. Rodrigo Jorge

FMRP-USP

Study justification:

Postoperative endophthalmitis is one of the most feared complications of intraocular surgery, and the use of 5% PI (povidone-iodine) alone has been shown to be effective in prophylaxis. Different methodologies in the application of PI eye drops have not been properly studied to define the most effective protocol in the application of PI in reducing bacterial conjunctival flora. Our study aims to evaluate the efficacy of 5% PI eye drops in reducing the bacterial flora in the conjunctival sac comparing the application of 3 drops at the times: 00min, 20min and 28min (time counted from the application of the first drop of PI) compared to the standard procedure, which corresponds to the application of only 1 drop of PI 2 minutes before any intraocular surgical procedure.

In this study, we intend to verify if the new protocol of prophylaxis against postoperative endophthalmitis will present a better efficacy in the reduction of bacterial conjunctival flora in relation to the standard treatment, comparing the effectiveness of the two methods in eliminating or reducing the number of microorganisms present in the conjunctival sac.

3. Objetives:

General

- To evaluate the efficacy of the application of 3 drops of 5% PI in the reduction of bacterial flora in the conjunctival sac for the prevention of postoperative endophthalmitis.

Specific

- To evaluate the efficacy of 5% PI eye drops in the quantitative and qualitative reduction of the conjunctival bacterial flora in the different protocols.

- Quantify the number of colonies in samples with bacterial growth.

 - Identify which bacteria grew in the culture media studied.

- Check the effects of the procedure on corneal thickness by pachymetry.

Risks and Benefits:

Patients, when included in the research, will not present risks due to postoperative complications, since no additional surgical procedure will be performed. Patients will be monitored at the Retina Section of the Department of Ophthalmology, School of Medicine of Ribeirão Preto (HC-FMRP-USP).

Patients under study may present an allergic reaction to eye drops, even though they have a negative personal history of iodine allergy. It is possible for patients to present corneal desepithelialization and conjunctival chemosis after use of the eye drops, a process that is reversible in 1-3 days and that rarely occurs with the 5% PI concentration.

Patients submitted to the analysis of conjunctival material by the Microbiology Laboratory will have the advantage of having their treatment better directed by the doctor who accompanies it in case of eventual endophthalmitis since it will be possible to know which microorganism is part of its conjunctival flora. Many of them will undergo ophthalmologic surgeries (such as cataract surgery and / or posterior vitrectomy) and may benefit from the information regarding their conjunctival flora, in relation to endophthalmitis prophylaxis. Some patients must undergo repeated intravitreal injections (every 6/8 weeks) and may also benefit from the information obtained in the study. If the 3 PI drops scheme shows to be more efficient, it will be applied and will benefit the patients of the study, as well as the other HCFMRP-USP patients.

4. Materials and methods:

Study design:

Prospective and randomized.

Selection of patients:

The study will include 40 patients attending the Retina and Vitreous Service of the Hospital das Clínicas of the Medical School of Ribeirão Preto scheduled for surgical procedures, at random, provided they meet the inclusion criteria and respect the exclusion criteria.

Inclusion criteria:

- Age: over 18 years of age.

- Absence of systemic infection or ocular infection during the study period.

- Absence of autoimmune diseases or immunosuppressive therapy.

- No use of antibiotics within the last 10 days preceding the procedure.

- Absence of eye surgery or trauma to the eye that will be involved in the last 30 days.

- No history of allergy to iodine and its derivatives.

- Signed free informed consent form (TCLE).

Exclusion Criteria:

- Intraocular surgery in the last 30 days.

- Use of antibiotics in the last 10 days preceding the procedure.

- Treatment with ionizing radiation in the face, skull and neck region.

- Diagnosis of Diabetes Mellitus.

- Systemic infection, ocular infection, blepharitis, ectropion, entropion or distriquiasis.

- Known abusive use of alcohol or drugs.

- Medical or psychological conditions that prevent the patient from completing the study or signing informed consent.

- Significant and uncontrolled disease that, in the opinion of the investigator, may exclude the patient from the study.

- Impediment or limited legal capacity.

- Participation in another clinical study in the last 30 days.

Study Procedures:

Patients eligible for admission, after being clarified and having signed the consent form, will be included in the study being randomly assigned to two groups by lottery.

In Group 1, which will be called the PI group, patients will receive 3 drops of 5% PI in the eye studied. In Group 2, which will be called the Control Group, the selected patients will receive only 1 drop of 5% PI.

In the PI Group, each patient will initially undergo pachymetry before the application of 5% PI eye drops. An initial pachymetry and biomicroscopic evaluation of the anterior segment shall be performed, then 1 drop of anesthetic eye drops of 0.5% Proximetacaine Hydrochloride shall be squeezed and a sample of the background material from the conjunctival sac will be collected with a sterile swab 5 minutes before application of the 1^st^ PI drop. The application of the first 5% PI drop will be done in time 00min, the 2^nd^ PI drop will be applied in time 20min and the 3^rd^ drop in time 28min. Two minutes after dribbling the 3rd. Drop of PI, that is, in time 30min, a second conjuntival swab sample will be collected.

Finally, after the collection of the 2^nd^ swab, the patient will perform a second pachymetry and a new biomicroscopic evaluation of the anterior segment by the slit lamp for follow-up and evaluation of some possible damage or adverse effect.

In the Control Group, patients will be submitted to the same procedure described above, except for replacing the 1st and 2nd drops of 5% PI with sterile saline solution 0.9% (SS 0.9%) in order to serve as a comparative group of the study.

Immediately before each drop of PI or SS 0.9% 1 drop of anesthetic eye drops of 0.5% Proximetacaine Hydrochloride is applied. Samples will be transferred immediately to the microbiology laboratory where they will be incubated in liquid thioglycollate medium (broth at 37 ° C) and in three solid culture media (chocolate agar, Trypcase Soy Agar with 5% ram blood and Agar Sabouraud at 37 ° C). The liquid medium should be peeled when it appears cloudy or after 5 days of incubation without turbidity to improve the sensitivity of the process.

STUDY FLOWCHART

PI group and Control Group


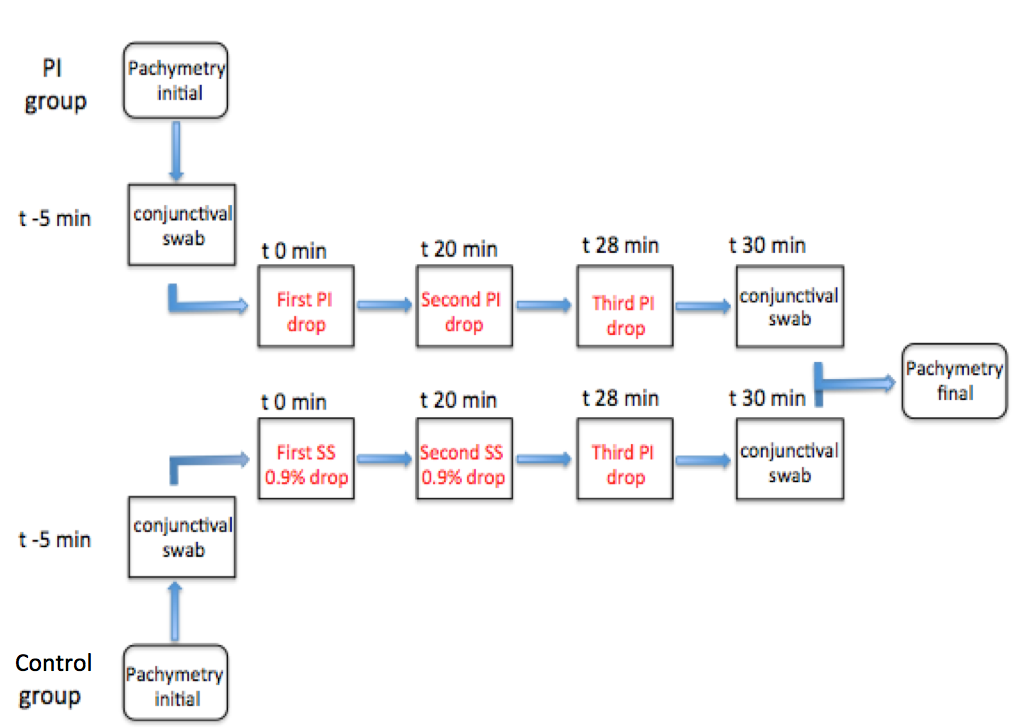


Statistical methods:

The pre vs. posttreatment comparison will be performed by Analysis of Variance, depending on the distribution presented by the variables analyzed. We will adopt the level of statistical significance 0.05.

5. References:

Aaberg TM Jr, Flynn HW Jr, Schiffman J, Newton J. Nosocomial acute-onset postoperative endophthalmitis survey; a 10-year review of incidence and outcomes. Ophthalmology 1998;105:1004–1010.

Apt L, Isenberg S, Yoshimori R, Paez JH. Chemical preparation of the eye in ophthalmic surgery. III: effect of povidone-iodine on the conjunctiva. Arch Ophthalmol 1984; 102:728–729.

Apt, L., Isenberg, S.J., Yoshimori, R., et al. Outpatient topical use of povidone–iodine in preparing the eye for surgery. Ophthalmology. 1989; 96:289–292.

Arantes, Tiago Eugênio Faria e et al. Flora bacteriana conjuntival após uso tópico de ciprofloxacino e gatifloxacino em cirurgia de catarata. Arq. Bras. Oftalmol. [online]. 2008, vol.71, n.2, pp. 191-196).

Bannerman TL, Rhoden DL, McAllister SK, et al. The source of coagulase-negative staphylococci in the Endophthalmitis Vitrectomy Study; a comparison of eyelid and intraocular isolates using pulsed-field gel electrophoresis; the Endophthalmitis Vitrectomy Study Group. Arch Ophthalmol 1997; 115:357–361.

Caldwell DR, Kastl PR, Cook J, Simon J. Povidone-iodine: it is efficacy as a preoperative conjunctival and periocular preparation. Ann Ophthalmol 1984; 16:577, 580.

Chase R C, Ellis P P. Iodophors and skin asepsis: Iodophors as skin antiseptics before ophthalmic surgery. Ann Ophthalmol. 1970;12:312–317.

Clinical ocular pharmacology. Jimmy D Bartlett and Siret D Jaanus.. Butterworth-Heinemann, 4th edition, 2001, p-33).

Ciulla TA, Starr MB, Masket S. Bacterial endophthalmitis prophylaxis for cataract surgery. An evidence-based update. Ophthalmology 2002;109:13–26.

Dereklis DL, Bufidis TA, Tsiakiu EP, Palassopoulos SI: Preoperative ocular disinfection by the use of povidone-iodine 5%. Arc Ophthalmol 1994; 72:627-630

Dickey JB, Thompson KD, Jay WM. Anterior chamber aspirate cultures after uncomplicated cataract surgery. Am J Ophthalmol. 1991;112(3):278-82. Comment in: Am J Ophthalmol. 1992;113(2):221-2.

Gershenfeld L. Povidone-iodine as a topical antiseptic. Am J Surg 1957; 94:938-9.

González Bandrés C, Carrilero Ferrer MJ, Buznego Suárez L, García Claramunt MA, Méndez Llata M, Paredes B, Moriche Carretero M. [Efficacy of topical povidone-iodine applied the day before cataract surgery to reduce conjunctival flora](http://www.ncbi.nlm.nih.gov/pubmed/11340513?ordinalpos=99&itool=EntrezSystem2.PEntrez.Pubmed.Pubmed_ResultsPanel.Pubmed_DefaultReportPanel.Pubmed_RVDocSum). Arch Soc Esp Oftalmol. 2001 Apr;76(4):229-34. Spanish.

Han, D.P., Wisniewski, S.R., Wilson, L.A., et al. Spectrum and susceptibilities of microbiologic isolates in the Endophthalmitis Vitrectomy Study. Am. J. Ophthalmol.122:1–17, 1996.

Hodge W, Biu D. Frequency of recovery of ciprofloxacin-resistant ocular isolates following topical cirpofloxacin. Invest Ophthalmol Vis Sci 1995;36:155.

Isenberg S, Apt L, Yoshimori R, Khwarg S. Chemical preparation of the eye in ophthalmic surgery. IV. Comparison of povidone-iodine on the conjuntiva with a prophylactic antibiotic. Arch Ophthalmol. 1985;103(9):1340-2.

Jager RD, Aiello LP, Patel SC, Cunningham ET Jr. Risks of intravitreous injection: a comprehensive review. Retina 2004; 24: 676-698.

Kaspar HM, Chang RT, Singh K, et al. Prospective randomized comparison of 2 different methods of 5% povidone- iodine applications for anterior segment intraocular surgery. Arch Ophthalmol 2005; 123:161–165

Kaspar HM, Chang RT, Shriver EM, et al. Three-day application of topical ofloxacin reduces the contamination rate of microsurgical knives in cataract surgery. A prospective randomized study. Ophthalmology 2004; 111:1352–1355.

[Mac Rae SM](http://www.ncbi.nlm.nih.gov/pubmed?term=), [Brown B](http://www.ncbi.nlm.nih.gov/pubmed?term=), [Edelhauser HF](http://www.ncbi.nlm.nih.gov/pubmed?term=). The corneal toxicity of presurgical skin antiseptics. Am J Ophthalmol. 1984 Feb;97(2):221-32.

Mah FS. Fourth-generation fluoroquinolones: new topical agents in the war on ocular bacterial infections. Curr Opin Ophthalmol 2004;15:316-20.

Mason JO 3rd, White MF, Feist RM, Thomley ML, Albert MA, Persaud TO, Yunker JJ, Vail RS. [Incidence of acute onset endophthalmitis following intravitreal bevacizumab (Avastin) injection.](http://www.ncbi.nlm.nih.gov/pubmed/18398358?ordinalpos=15&itool=EntrezSystem2.PEntrez.Pubmed.Pubmed_ResultsPanel.Pubmed_DefaultReportPanel.Pubmed_RVDocSum) Retina. 2008 Apr;28(4):564-7.

Maumenee A E, Michler R C. Sterility of the operative field after ocular surgery. Pac Coast Oto-Ophthalmol Soc. 1951;32:172–183

Moshfeghi DM, Kaiser PK, Scott IU, et al. Acute endophthalmitis following intravitreal triamcinolone acetonide injection. Am J Ophthalmol. 2003;136:791-796.

# [**Moss JM**](http://www.ncbi.nlm.nih.gov/pubmed?term=), [**Sanislo SR**](http://www.ncbi.nlm.nih.gov/pubmed?term=), [**Ta CN**](http://www.ncbi.nlm.nih.gov/pubmed?term=). A prospective randomized evaluation of topical gatifloxacin on conjunctival flora in patients undergoing intravitreal injections. **Ophthalmology.** 2009 Aug;116(8):1498-501.

Sakamoto T, Enaida H, Kubota T, et al. Incidence of acute endophthalmitis after triamcinolone-assisted pars plana vitrectomy. Am J Ophthalmol. 2004;138:137-138.

Samad A, Solomon LD, Miller MA, Mendelson J. Anterior chamber contamination after uncomplicated phacoemulsification and intraocular lens implantation. Am J Ophthalmol. 1995;120(2):143-50.

Seppala H, Al-Juhaish M, Jarvinen H, et al. Effect of prophylactic antibiotics on

antimicrobial resistance of viridans streptococci in the normal flora of cataract surgery patients. J Cataract Refract Surg 2004;30:307-15.

Shelanski HÁ, Shelanski MV. PVP-iodine: history, toxicity and therapeutic uses. J Int Coll Surg 1956;25:727-34.

Sousa LB. Prevenção da infecção na cirurgia intra-ocular. In: Sousa LB, Freitas D, Höfling-Lima AL, Nishiwaki-Dantas MC, editores, Manual de prevenção da infecção nos procedimentos oftalmológicos. São Paulo: Lemos; 2003. p.129-34

[Speaker MG](http://www.ncbi.nlm.nih.gov/pubmed?term=), [Milch FA](http://www.ncbi.nlm.nih.gov/pubmed?term=), [Shah MK](http://www.ncbi.nlm.nih.gov/pubmed?term=), [Eisner W](http://www.ncbi.nlm.nih.gov/pubmed?term=), [Kreiswirth BN](http://www.ncbi.nlm.nih.gov/pubmed?term=). Role of external bacterial flora in the pathogenesis of acute postoperative endophthalmitis. Ophthalmology. 1991 May;98(5):639-49; discussion 650.

Speaker MG, Menikoff JA: Prophylaxis of endophthalmitis with topical povidone-iodine. Ophthalmol 98:1769-1775, 1991

Speaker MG, Milch FA, Shah MK, Eisner W, et al: Role of external bacterial flora in the pathogenesis of acute postoperative endophthalmitis survey. A 10-year review of incidence and outcomes. Ophthalmol 105:1004-1010, 1998

Ta CN, Chang RT, Singh K, Egbert PR, Shriver EM, Blumenkranz MS, et al. Antibiotic resistance patterns of ocular bacterial flora. A prospective study of patients undergoing anterior segment surgery. Ophthalmology. 2003; 110(10):1946-51.

Ta CN,  Singh K,  Egbert PR,  Kaspar HM. Prospective comparative evaluation of povidone–iodine (10% for 5 minutes versus 5% for 1 minute) as prophylaxis for ophthalmic surgery. Journal of Cataract & Refractive Surgery, Volume 34, Issue 1, January 2008, Pages 171-172

[Trinavarat A](http://www.ncbi.nlm.nih.gov/pubmed?term=), [Atchaneeyasakul LO](http://www.ncbi.nlm.nih.gov/pubmed?term=), [Nopmaneejumruslers C](http://www.ncbi.nlm.nih.gov/pubmed?term=), [Inson K](http://www.ncbi.nlm.nih.gov/pubmed?term=)**. Reduction of endophthalmitis rate after cataract surgery with preoperative 5% povidone-iodine. Dermatology**, Jan 2006; 212 Suppl 1: 35-40.

Walker CB, Claone CM. Incidence of conjunctival colonization by bacteria capable of causing postoperative endophthalmitis. J R Soc Med 1986; 79: 520-521.

Westfall AC, Osborn A, Kuhl D, Benz MS, Mieler WF, Holz ER. **Acute endophthalmitis incidence: intravitreal triamcinolone. Arch Ophthalmol**, Aug 2005; 123: 1075-7

Wu PC, Li M, Chang SJ, Teng MC, Yow SG, Shin SJ, Kuo HK. [Risk of endophthalmitis after cataract surgery using different protocols for povidone- iodine preoperative disinfection.](http://www.ncbi.nlm.nih.gov/pubmed/16503776?ordinalpos=49&itool=EntrezSystem2.PEntrez.Pubmed.Pubmed_ResultsPanel.Pubmed_DefaultReportPanel.Pubmed_RVDocSum). J Ocul Pharmacol Ther. 2006 Feb;22(1):54-61.
